# A High-Resolution Crystallographic Study of Cytochrome c6: Structural Basis for Electron Transfer in Cyanobacterial Photosynthesis

**DOI:** 10.3390/ijms26020824

**Published:** 2025-01-19

**Authors:** Botao Zhang, Yuancong Xu, Shuwen Liu, Sixu Chen, Wencong Zhao, Zhaoyang Li, Junshuai Wang, Weijian Zhao, Heng Zhang, Yuhui Dong, Yong Gong, Wang Sheng, Peng Cao

**Affiliations:** 1 College of Chemistry and Life Science, Beijing University of Technology, Beijing 100124, China; zhbbt@emails.bjut.edu.cn (B.Z.); xuyuancong@bjut.edu.cn (Y.X.); lsw75097@163.com (S.L.); sixuchen@emails.bjut.edu.cn (S.C.); wencongzhao@emails.bjut.edu.cn (W.Z.); zhaoyangli@emails.bjut.edu.cn (Z.L.); wjunshuai@emails.bjut.edu.cn (J.W.); zhaowj@emails.bjut.edu.cn (W.Z.); 2 Beijing Synchrotron Radiation Facility, Institute of High Energy Physics, Chinese Academy of Sciences, Beijing 100049, China; zhangheng@ihep.ac.cn (H.Z.); dongyh@ihep.ac.cn (Y.D.); yonggong@ihep.ac.cn (Y.G.); 3 Institute of Matter Science, Beijing University of Technology, Beijing 100124, China

**Keywords:** cytochrome c6, photosystem I, cyanobacteria, electron transfer, X-ray crystallography, dimerization

## Abstract

Cyanobacterial cytochrome c6 (Cyt c6) is crucial for electron transfer between the cytochrome b6f complex and photosystem I (PSI), playing a key role in photosynthesis and enhancing adaptation to extreme environments. This study investigates the high-resolution crystal structures of Cyt c6 from *Synechococcus elongatus* PCC 7942 and *Synechocystis* PCC 6803, focusing on its dimerization mechanisms and functional implications for photosynthesis. Cyt c6 was expressed in *Escherichia coli* using a dual-plasmid co-expression system and characterized in both oxidized and reduced states. X-ray crystallography revealed three distinct crystal forms, with asymmetric units containing 2, 4, or 12 molecules, all of which consist of repeating dimeric structures. Structural comparisons across species indicated that dimerization predominantly occurs through hydrophobic interactions within a conserved motif around the heme crevice, despite notable variations in dimer positioning. We propose that the dimerization of Cyt c6 enhances structural stability, optimizes electron transfer kinetics, and protects the protein from oxidative damage. Furthermore, we used AlphaFold3 to predict the structure of the PSI-Cyt c6 complex, revealing specific interactions that may facilitate efficient electron transfer. These findings provide new insights into the functional role of Cyt c6 dimerization and its contribution to improving cyanobacterial photosynthetic electron transport.

## 1. Introduction

Cyanobacteria exhibit remarkable adaptability to extreme environments, such as high salinity, intense light, fluctuating temperatures, and nutrient limitations. This adaptability is primarily due to their highly efficient photosynthetic systems, where cytochrome c6 (Cyt c6) serves as a crucial component ensuring effective electron transport [1]. Cyt c6 is a small, water-soluble protein (~12 kDa) that contains a heme group as its active center. As an essential electron carrier, Cyt c6 mediates between the cytochrome b6f complex and photosystem I (PSI), enabling electron transfer that is fundamental for sustaining photosynthesis by maintaining the continuous flow of electrons through the photosynthetic chain. Specifically, Cyt c6 accepts electrons from cytochrome b6f and transfers them to PSI, driving the conversion of light energy into chemical energy [2]. The redox potential of Cyt c6, along with its binding affinity to both PSI and the cytochrome b6f complex, is critical for determining the efficiency of electron transfer within this chain [3]. Recent advances have explored the immobilization of PSI on electrodes for developing green, sustainable semi-artificial semiconductor systems, with Cyt c6 enhancing electron transfer and photoelectric conversion efficiency [4,5].

In cyanobacteria, Cyt c6 is the predominant electron carrier, replacing plastocyanin (Pc), which is often absent or less prevalent in cyanobacteria. In contrast, Pc is the primary electron carrier in green algae under normal conditions, whereas in copper-deficient environments, algae utilize Cyt c6, demonstrating its adaptive flexibility [6]. In heterocysts of *Anabaena* sp. PCC 7120, Cyt c6 acts as the primary soluble electron donor, even with sufficient copper, indicating a unique role in heterocyst function [7]. The introduction of algal Cyt c6 into transgenic *Arabidopsis* or tobacco significantly enhances photosynthetic efficiency and growth, demonstrating potential applications in improving bioenergy crop productivity [8,9]. Additionally, the Cyt c6 family includes several homologs, such as Cyt c6A, which is found in plants and green algae but does not participate in photosynthetic electron transfer. In contrast, Cyt c6B and Cyt c6C, found in cyanobacteria, are involved in electron transfer within the photosynthetic process. These homologs exhibit functional specialization, with redox potentials approximately 200 mV lower than that of Cyt c6 [10,11,12,13].

Although class I c-type cytochromes are generally considered monomeric, structural studies in photosynthetic algae have provided valuable insights into the oligomerization behavior of Cyt c6. Previous crystallographic analyses indicate that Cyt c6 forms weak dimers or oligomers. The high-resolution structure of Cyt c6 from *Chlamydomonas reinhardtii* (PDB code: 1CYJ) suggests functional dimerization that may optimize electron transfer efficiency [14]. Similarly, weak dimerization mediated by hydrophobic interactions has been observed in *Phormidium laminosum* Cyt c6 (PDB code: 2V08), indicating its biological relevance for maintaining stability under specific conditions [15]. Other studies, including *Hizikia fusiformis* (PDB code: 2ZBO), *Thermosynechococcus vestitus* BP-1 (PDB code: 6TSY) and *Arabidopsis thaliana* (PDB codes: 2V07, 2CE0, 2CE1), have revealed dimeric and oligomeric states in Cyt c6 [15,16,17,18]. The oligomerization state can also be influenced by environmental factors, with domain swapping during refolding further impacting oligomerization [19,20]. These findings imply a general propensity for oligomerization, but the precise structural and functional roles of these oligomeric states remain unclear. Furthermore, recent cryo-electron microscopy (cryo-EM) studies have explored interactions between Pc or Cyt c6 and PSI [21,22]. However, the exact binding site of Cyt c6 on PSI remains to be determined.

In this study, we employed a dual-plasmid co-expression strategy to co-express Cyt c6 with the heme synthesis cluster (pEC86) in *Escherichia coli*. Cyt c6 proteins from *Synechococcus elongatus* sp. PCC 7942 (*S. elongatus* PCC 7942) and *Synechocystis* sp. PCC 6803 (*S.* PCC 6803) were successfully expressed and purified. High-resolution X-ray diffraction was used to analyze three crystal structures of Cyt c6. To further understand the interaction, AlphaFold3 was used to predict the PSI-Cyt c6 complex structure, providing additional insights into the electron transfer mechanism. These findings advance our understanding of Cyt c6’s structure and function, shedding light on how cyanobacteria adapt their photosynthetic processes to extreme environments.

## 2. Results

### 2.1. Preparation and Characterization of Cyt c6 Proteins

**Figure 1:**
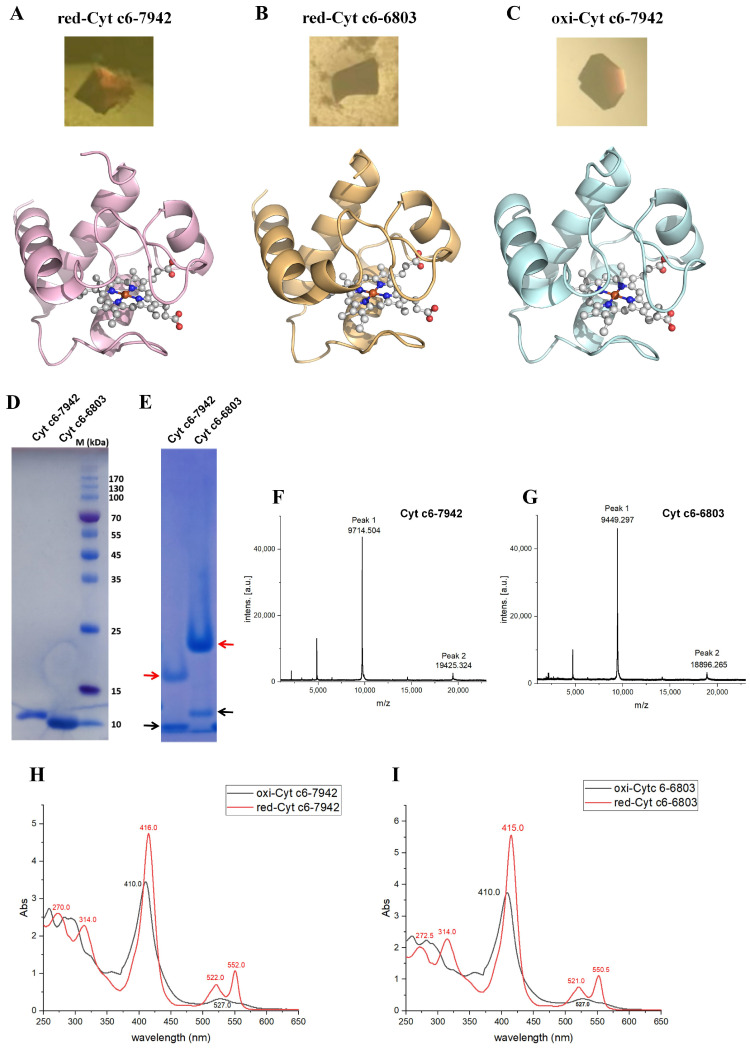
Crystallization and characterization of Cyt c6 proteins. (**A**–**C**) Structural models and crystal images of red-Cyt c6-7942, red-Cyt c6-6803, and oxi-Cyt c6-7942. The ball-and-stick models highlight the bound heme groups. (**D**) SDS-PAGE analysis of purified Cyt c6-6803 and Cyt c6-7942. (**E**) Blue native PAGE analysis of Cyt c6-6803 and Cyt c6-7942. Potential positions of dimer and monomer are indicated by red and black arrows, respectively. (**F**,**G**) MALDI-TOF mass spectrometry results for Cyt c6-7942 (**F**) and Cyt c6-6803 (**G**). (**H**,**I**) UV-vis spectroscopy analysis of the absorbance properties of Cyt c6-7942 (**H**) and Cyt c6-6803 (**I**).

To enhance expression and ensure correct heme incorporation of Cyt c6 in *Escherichia coli*, a co-expression strategy was employed using the pEC86 plasmid, which encodes the ccmA-H gene cluster essential for heme maturation in the periplasm [23]. The reduced Cyt c6 proteins were generated using *β*-mercaptoethanol, while oxidized forms were prepared by adding potassium ferricyanide. Three distinct crystal forms of Cyt c6 were obtained using the sitting drop vapor diffusion method, yielding characteristic red crystals indicative of heme incorporation (Table S1). High-resolution X-ray diffraction data determined the three-dimensional structures of reduced Cyt c6-7942 (red-Cyt c6-7942) at 1.7 Å resolution, reduced Cyt c6-6803 (red-Cyt c6-6803) at 1.9 Å resolution, and oxidized Cyt c6-7942 (oxi-Cyt c6-7942) at 1.4 Å resolution (Figure 1A–C; the crystallographic statistics shown in Table S2). Due to the complexity and unpredictability of crystallization, we were unable to obtain the crystals of oxidized Cyt c6-6803.

SDS-PAGE analysis confirmed the high purity of Cyt c6 proteins from both cyanobacterial sources, with distinct bands indicating differences in physicochemical properties between Cyt c6-7942 and Cyt c6-6803 (Figure 1D). Blue native PAGE (4–13%) revealed the presence of both dimers and monomers for Cyt c6-7942 and Cyt c6-6803 in solution (Figure 1E). MALDI-TOF mass spectrometry revealed that both Cyt c6-7942 and Cyt c6-6803 primarily existed as monomers, with molecular masses of 9714.504 and 9449.297 (Peak1), respectively. Minor peaks corresponding to potential dimeric forms (Cyt c6-7942: 19,425.324; Cyt c6-6803: 18,896.265; Peak2) suggested the possible presence of dimeric species in solution (Figure 1F,G). UV–visible absorption spectroscopy was used to characterize the redox properties of Cyt c6 variants. Both Cyt c6-7942 and Cyt c6-6803 exhibited characteristic spectral changes upon oxidation, including a blue shift and decreased intensity at 415 nm compared to the reduced form (Figure 1H,I). In the reduced form, peaks at 522 nm and 550 nm were replaced by a broader peak at 525 nm in the oxidized form, and a peak at 314 nm disappeared upon oxidation. These spectral changes were consistent with previously reported data [16], indicating that Cyt c6 undergoes similar redox-dependent spectral shifts, regardless of the cyanobacterial source.

### 2.2. Crystal Structural Analysis

**Figure 2:**
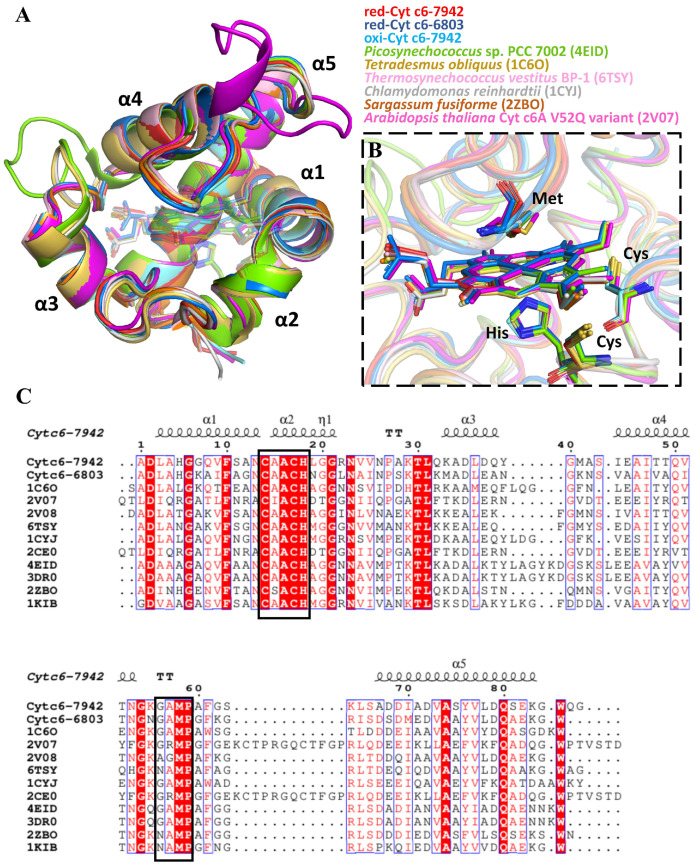
Comparative structural and sequence analysis of Cyt c6 in photosynthetic organisms. (**A**) Structural alignment of red-Cyt c6-7942, red-Cyt c6-6803, oxi-Cyt c6-7942, and previously reported Cyt c6/C6A structures from *Prochlorococcus* sp. PCC 7002 (PDB code: 4EID), *Tetradesmus obliquus* (PDB code: 1C6O), *Thermosynechococcus vestitus* BP-1 (PDB code: 6TSY), *Chlamydomonas reinhardtii* (PDB code: 1CYJ), *Sargassum fusiforme* (PDB code: 2ZBO), and *Arabidopsis thaliana* (PDB code: 2V07). (**B**) An enlarged view of (**A**), highlighting the amino acid residues involved in direct heme binding shown as sticks. (**C**) Sequence alignment of Cyt c6 and Cyt c6A. The ESPript 3.0 server (http://espript.ibcp.fr/ESPript/ESPript/, accessed on 1 November 2024) was used to output the alignment. Conserved residues, including the CXXCH motif and an ancient structural motif near the methionine axial ligand, are highlighted with black boxes. Colours represent residue similarity: red background for identical residues, red text for strongly similar residues. Blue frame indicates that over 70% of residues are similar in physico-chemical properties. The secondary structure is annotated with five α-helices.

The crystal structures of Cyt c6 from both species revealed a conserved fold, consisting of five α-helices, a bound heme group, and loop regions, which closely resembled previously characterized Cyt c6 proteins from other species (Figure 2A) [14,15,16,17,18]. The heme group was embedded in a hydrophobic pocket, with two propionate groups extending outward, allowing for flexibility in the binding environment. This flexibility influenced the hydrogen bonding network around the heme-binding site, consistent with previous NMR observations [24]. The heme was covalently linked to two cysteine residues via thioether bonds, and the central iron atom was axially coordinated by methionine and histidine residues, maintaining consistent coordination geometry in both oxidized and reduced states (Figure 2B). Compared with red-Cyt c6-7942, the global the root-mean-square deviation (RMSD) values of a single monomer across multiple species range from 0.210 to 0.648 Å (Table S3). By extracting the RMSD values for each residue from the alignment results, we observed that residues with lower RMSD values indicate conserved three-dimensional positions (Figure S1). In contrast, residues with higher RMSD values show a lack of conservation between species.

Three structures were solved in the *P*2_1_2_1_2_1_ and *P*2_1_ space groups, with asymmetric units containing 2, 4, or 12 molecules, each exhibiting distinct packing arrangements (Figure S2). Crystal packing analysis indicated that the dimers in red-Cyt c6-7942 and red-Cyt c6-6803 crystals formed higher-order assemblies. The tetramers and dodecamers were essentially composed of repeating dimeric units. Upon extracting and aligning these dimers, their positions were nearly identical, with a RMSD of 0.259 Å between the two distinct dimers in the Cyt c6-7942 tetramer (Figure S2B). Structural comparison of the peripheral and central dimers within the Cyt c6-6803 dodecamer also revealed consistency, with RMSD values ranging from 1.330 to 2.149 Å (Figure S2D).

Minimal differences were observed between the reduced and oxidized forms, with an RMSD of 0.446 (Figure S2F), indicating that heme coordination remains stable throughout redox transitions, consistent with the previous report [17,25]. Structural comparisons between red-Cyt c6-7942 and oxi-Cyt c6-7942 revealed some differences in surface amino acids, particularly charged residues, despite the largely similar positions of heme-binding pocket residues. In red-Cyt c6-7942, Asp69 formed additional hydrogen bonds with His5, stabilizing the connection between two α-helices. Additionally, the hydrogen bond between Asn23 and Asn26, along with the interaction between Thr30 and Gln32, result in a more compact structure. Analysis of the RMSD values for each residue between the oxidized and reduced forms of Cyt c6-7942 (Figure S1) reveals some flexibility in the side chains of residues located on the protein surface, including Gln8, Arg22, Glu45, Thr48, Lys64, and Glu82. Further investigation using surface potential maps of both reduced and oxidized Cyt c6 shows that most of the negatively charged residues are consistent, in line with previous studies [24]. However, noticeable differences in the surface potential map are observed near the acidic patch (Asp68, Asp69, Asp72, and Asp2 in Cyt c6-7942), which can be attributed to the flexibility of the N-terminus (Figure S3E).

### 2.3. Structural Analysis of the Dimerization Interface

We used PISA (https://www.ebi.ac.uk/pdbe/pisa/, accessed on 15 November 2024) to characterize the aggregation state of our structures. The results indicated that the red-Cyt c6-7942 forms a stable dimer. The dimerization is energetically favorable, with an interaction free energy of −54.6 kcal/mol. However, the dissociation energy of 0.7 kcal/mol implies that the dimer is weakly stable and may dissociate in specific conditions. The two chains interact significantly, as suggested by the buried surface area and the inclusion of two heme cofactors in the assembly.

**Figure 3:**
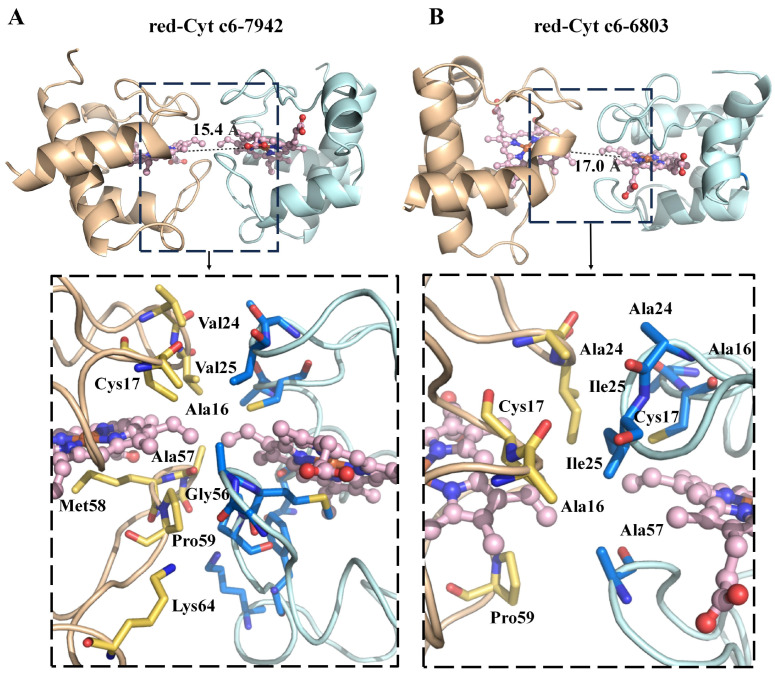
Dimeric interfaces of red-Cyt c6-7942 (**A**) and red-Cyt c6-6803 (**B**). The proteins are shown as cartoon representations. Heme groups are depicted as ball-and-stick models (light pink), while amino acid residues involved in the dimeric interaction interface are shown as stick models. The Fe–Fe distances between the heme groups are represented by black dashed lines, with their values labeled. The key interface residues are magnified, shown in the dashed boxes below.

Both Cyt c6-7942 and Cyt c6-6803 exhibited a “crevice-to-crevice” dimerization mode, resembling cytochrome dimerization patterns observed in previous studies [14,15,16,25]. The dimerization interface was primarily stabilized by hydrophobic interactions involving conserved residues, which explains the consistent dimerization tendency observed across different species (Figure 2C). Specifically, the key residues involved included Ala16, Val24, Val25, Gly56, Ala57, Met58, Pro59 and Lys64 for Cyt c6-7942 (Figure 3A). These interacting residues are part of the highly conserved sequence regions of Cyt c6, indicating their critical role in maintaining the structural integrity of the dimeric form. Additionally, the oxidized and reduced forms of Cyt c6-7942 exhibited highly consistent dimeric interactions.

**Figure 4:**
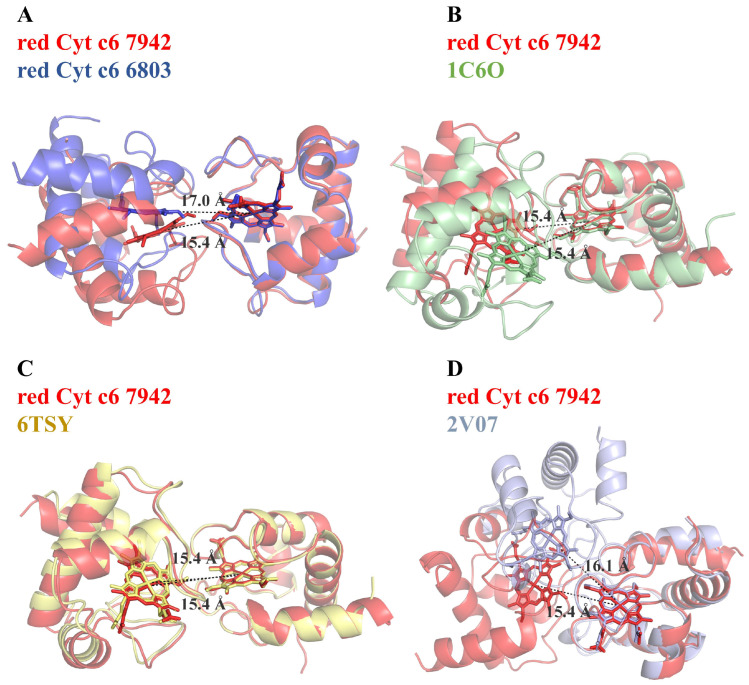
Comparative analysis of dimeric Cyt c6 structures from different species. The red-Cyt c6-7942 structure is compared with Cyt c6-6803 (**A**) and previously reported dimeric structures from *Tetradesmus obliquus* Cyt c6 (PDB code: 1C6O; (**B**)) [25], *Thermosynechococcus vestitus* BP-1 Cyt c6 (PDB code: 6TSY; (**C**)) [16], and *Arabidopsis thaliana* Cyt c6A (PDB code: 2V07; (**D**)) [15]. The red-Cyt c6-7942 structure is highlighted in red, with one monomeric chain of each dimer superimposed for comparison. Dashed lines indicate the Fe–Fe distances between heme groups.

The dimer interface of Cyt c6-6803 showed significant differences compared to that of Cyt c6-7942 (Figure 3B and Figure 4A), being less extensive and lacking contributions from residues near the methionine axial ligand. Key residues involved in the dimerization of Cyt c6-6803 included Ala16, Cys17, Ala24, Ile25 and Pro59. Additionally, the distance between the iron atoms of the two hemes in the Cyt c6-6803 dimer was 17.0 Å, compared to 15.4 Å in Cyt c6-7942 (Figure 3B). Notably, these Fe–Fe distances fall within the typical range for electron transfer, which is between 10 and 25 Å [26,27].

### 2.4. Comparative Analysis of Dimeric Structures

A comparative analysis was performed to evaluate the dimeric states of Cyt c6 observed in this study with previously reported structures (Figure 4). The results indicate that the Fe–Fe distances and interaction interfaces are highly conserved across different species, highlighting the crucial role of dimerization in Cyt c6’s function in electron transport. The Cyt c6-6803 dimer exhibited a larger Fe–Fe distance and weaker dimer interactions compared to other Cyt c6 dimers. Notably, when one monomer of Cyt c6-6803 was superimposed onto Cyt c6-7942, the other monomer exhibited a 20° rotation relative to Cyt c6-7942, indicating a significant difference in dimer assembly (Figure 4A). Despite the increased spacing, the hemes remained properly aligned, suggesting a structural flexibility in the dimers.

The interface areas between the dimeric proteins in Cyt c6-6803 and Cyt c6-7942 were consistent with previously reported values for similar dimers, ranging from 248 Å^2^ to 317 Å^2^. The distances between the iron atoms of the heme groups fell within the range of 15.4–17.5 Å, consistent with known dimeric interactions. These observations suggest that dimerization is an intrinsic feature of Cyt c6, driven by the structural characteristics of the heme pocket. Analysis of the amino acid composition of the dimer interfaces revealed several conserved residues, including the CXXCH motif and a structural motif near the methionine axial ligand (Figure 2C). These findings indicate that hydrophobic interactions predominantly drive dimerization, with conserved residues playing a key role in dimer formation across cyanobacterial species.

While dimeric interactions showed general consistency, variations were observed among different species. After performing comparative analysis of the dimers from various species to red-Cyt c6-7942, the RMSD values ranged from 0.752 to 12.643 (Table S4). The dimeric structure of Cyt c6-7942 closely resembled that of *Thermosynechococcus vestitus* BP-1 (PDB code: 6TSY; Figure 4C). In comparison to *Tetradesmus obliquus* (PDB code: 1C6O), superimposing one monomer revealed a 10° rotation of the other monomer, further highlighting species-specific structural variations. However, the heme distances remained identical to those in Cyt c6-7942 (Figure 4B). Furthermore, the Cyt c6A structure (PDB code: 2V07) contains an extended sequence (Lys66–Pro77) and exhibits the most significant differences in dimeric interactions. Superimposing one monomer of *Arabidopsis thaliana* Cyt c6A onto Cyt c6-7942 resulted in an approximate 90° rotation of the other monomer, bringing them closer together (Figure 4D). Notably, Cyt c6A does not participate in photosynthetic electron transfer, emphasizing its dimeric structural divergence from the Cyt c6 proteins of cyanobacteria.

### 2.5. AlphaFold3 Prediction of PSI-Cyt c6 Complex

**Figure 5:**
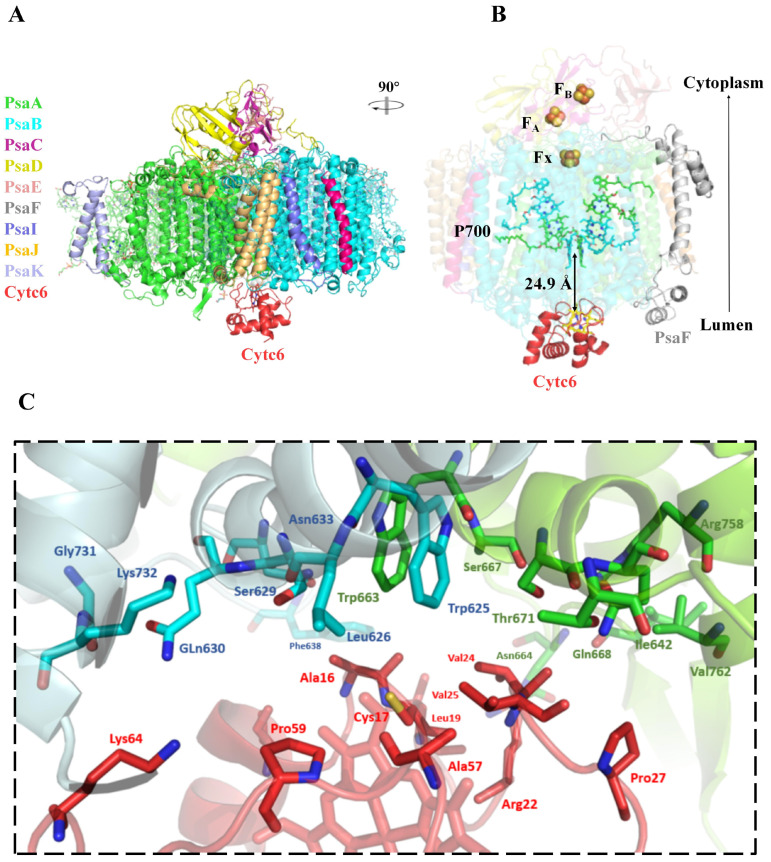
AlphaFold3-predicted structure of the PSI-Cyt c6 complex from *S. elongatus* PCC 7942. (**A**) Overall structure shown in cartoon representation. (**B**) The PSI-Cyt c6 electron transport chain, with the arrow indicating the direction of electron flow. (**C**) Residues forming the interaction interface between Cyt c6 and PsaA-PsaB, displayed as stick models. Cyt c6 is shown in red, PsaA in green, and PsaB in blue.

To gain deeper insights into the interaction between Cyt c6 and PSI, we employed AlphaFold3 [28] to predict the structure of the PSI-Cyt c6 complex from *S. elongatus* PCC 7942. During the prediction process, the inclusion of the heme molecule enhanced the reliability of the results. The predicted model was compared with the structure of monomeric PSI (PDB code: 6KIF) [29] and our experimentally determined Cyt c6 structure. The RMSD values of 0.492 Å for PSI and 0.408 Å for Cyt c6 indicated high structural similarity between the predicted and experimental models, supporting the reliability of the predicted PSI-Cyt c6 complex (Figure 5A).

In our model, Cyt c6 docked into a concave region on the lumenal side of PSI, primarily interacting with the PsaA and PsaB subunits. The heme group of Cyt c6 was oriented towards the PSI reaction center (Figure 5B), suggesting a plausible electron transfer pathway from Cyt c6 to PSI. The interaction was largely stabilized by hydrophobic contacts involving residues near the heme crevice (Figure 5C), consistent with previously observed interaction patterns in similar PSI complexes [30,31]. Specifically, the residues Ser11, Ala16, Cys17, Leu19, Arg22, Val24, Val25, Pro27, Ala57, Pro59, and Lys64 from Cyt c6 were found to interact with Ile642, Asn644, Trp663, Ala664, Ser667, Gln668, Thr671, Arg758, and Val762 of PsaA, as well as Trp625, Leu626, Ser629, Gln630, Asn633, Phe638, Gly731, and Lys732 of PsaB. No direct interaction between PsaF and Cyt c6 was observed, consistent with previous findings suggesting that PsaF is not essential for PSI function in cyanobacteria [32]. In contrast to Cyt c6, Pc interacts with PsbF through strong electrostatic interactions [21,22], implying their distinct modes of binding to PSI in the electron transfer process (Figure S3 and Figure 4).

## 3. Discussion

Our findings demonstrate that Cyt c6 from both oxidized and reduced states of *S. elongatus* PCC 7942 and *S* sp. PCC 6803 consistently forms dimers across all three crystal forms (Figure S2). While there are differences in the number of molecules 2, 4 or 12 in each asymmetric unit, structural analyses indicate that these dimeric units are primarily formed around the heme crevice through hydrophobic interactions within a conserved motif. This “crevice-to-crevice” dimerization mode likely serves to protect the heme active center, reducing exposure to oxidative damage and ensuring functional integrity. Comparisons with prior studies reveal similar dimerization patterns across cyanobacterial species, with conserved residues contributing to the dimer interface and Fe–Fe distances of approximately 15–17 Å between hemes, despite obvious variations in their relative positions (Figure 4).

The dynamic equilibrium between monomeric and dimeric forms may provide the flexibility necessary for efficient electron transport and adaptation to environmental changes. Previous reports have suggested that the oligomerization state of Cyt c6 can be influenced by environmental factors, with domain swapping during protein refolding playing a key role in modulating its oligomerization state [19,20]. In addition to dimers, trimers and higher-order polymerization can also be observed [16,19]. These various structural features likely enable Cyt c6 to dynamically adjust its electron transfer activity, optimizing photosynthetic efficiency in fluctuating cellular conditions. The biological significance of Cyt c6 dimerization, oligomerization and domain swapping may lie in its ability to enhance protein stability, optimize electron transfer kinetics, and protect the heme center from oxidative damage—traits that are especially advantageous in the extreme environments inhabited by cyanobacteria.

Small structural differences between red-Cyt c6-7942 and oxi-Cyt c6-7942 suggest insights into the functional mechanism of photosynthetic electron transfer. In the reduced form, non-heme-binding sites contribute to a more compact structure, which helps stabilize the dimeric configuration. In contrast, the oxidized form undergoes structural adjustments to accommodate the oxidized heme, providing the flexibility required for efficient electron transfer and subsequent dissociation from PSI. This rapid structural transition may facilitate flexible and effective electron transfer through Cyt c6. Compared to the previous report, which described the crystal structure of reduced Cyt c6 from *Tetradesmus obliquus* as forming monomeric aggregates [25], we speculate that the crystallization conditions, which lacked ammonium sulfate and instead used PEG, may contribute to the formation of the monomeric crystallization form in the monomer-dimer mixture.

From an evolutionary perspective, plastocyanin (Pc) has become the primary electron carrier to PSI in higher plants, while Cyt c6 has been lost during the evolutionary process [33]. Despite significant differences in primary and tertiary structures, the redox potentials of Cyt c6’s heme and Pc’s copper center are nearly identical, reflecting their functional roles in photosynthetic electron transport (Figure S3). Unlike Pc, which remains strictly monomeric, Cyt c6 has a propensity for dimerization, suggesting unique evolutionary adaptations to cyanobacterial ecological niches. The dimerization of Cyt c6 appears to be mediated by an evolutionarily conserved motif near the methionine axial ligand, represented as GAMP in cyanobacteria and green algae, or NAMP in other species, such as brown algae (Figure 2C). This methionine-containing motif is critical for heme coordination and likely influences dimerization, conferring evolutionary advantages for stability and electron transfer.

The predicted PSI-Cyt c6 complex model by AlphaFold3 positions Cyt c6 optimally for efficient electron donation to PSI, specifically targeting the P700 chlorophyll dimer from PsaA and PsaB subunits. The interactions between Cyt c6 and PSI primarily involve a hydrophobic binding interface with the PsaA-PsaB subunits, and residues mediating this interaction overlap with those responsible for dimerization in the crystal structure (Figure 5C). This suggests that weak dimeric interactions would be disrupted during PSI binding, exposing hydrophobic residues critical for electron transfer. The absence of interaction between Cyt c6 and the PsaF subunit in our model aligns with sequence alignments, which show that cyanobacterial PsaF lacks the positively charged residues essential for such interactions in higher plants (Figure S4). Studies on *S*. PCC 6803 PsaF deletion mutants support this opinion, demonstrating that PsaF is not essential for PSI electron transfer in cyanobacteria [32]. Cyanobacteria primarily rely on Cyt c6 as an electron carrier, whereas Pc plays a more prominent role in higher plants, with the PsaF-Pc interaction further optimizing this electron transfer mechanism.

Our predicted model aligns with previous molecular dynamics simulations [34]. However, a recent study resolved the structure of *Thermosynechococcus elongatus* BP-1 PSI complexed with ferredoxin and loosely bound Cyt c6 [22]. In comparison, the predicted Cyt c6 binding site in our model is located far from the loosely bound Cyt c6 density reported in that study (Figure S5). We propose that Cyt c6 functions by transitioning from a dimer-protected state (our crystal structures) to a temporary docking state [22], and ultimately to an electron transfer-ready state (our prediction structures) [30,31,35]. This dynamic conversion mechanism highlights the versatility of Cyt c6 in balancing heme protection with efficient electron transfer, reinforcing its pivotal role in cyanobacterial photosynthetic electron transport. The detailed structural analysis provides valuable insights into the molecular mechanisms underlying electron transfer in cyanobacterial photosynthesis, emphasizing Cyt c6’s adaptability as an electron carrier and its critical contribution to maintaining photosynthetic efficiency under extreme environments. However, the predicted binding sites still require further experimental validation using methods such as cross-linking experiments or co-immunoprecipitation (Co-IP) studies.

## 4. Materials and Methods

### 4.1. Expression and Purification of Cyt c6

The genes encoding Cyt c6 from *S. elongatus PCC* 7942 (UniProt ID: P25935) and *S.* PCC 6803 (UniProt ID: P46445) were cloned into the pET22b expression vector, including an N-terminal pelB signal peptide for periplasmic targeting. Following the method outlined in the references [23,36], plasmids pET22b-6803 and pET22b-7942 were co-transformed with the pEC86 plasmid into chemically competent *Escherichia coli* BL21 (DE3) for protein overexpression. The pEC86 plasmid is commonly used in *Escherichia coli* for bacterial cytochrome c-type protein expression. Small-scale overnight cultures were inoculated into 1.7 L of LB medium containing 34 μg/mL chloramphenicol and 100 μg/mL carbenicillin, and cultivated at 150 rpm and 30 °C. Protein expression was induced by adding 1 mM IPTG and 1 mM ferric chloride. Cells were harvested by centrifugation at 7000 rpm for 10 min, resuspended in buffer containing 20 mM Tris pH 8.0, 20 mM imidazole, and 500 mM NaCl, and stored at −80 °C.

Cell lysis was performed using high-pressure homogenization with the addition of 1 mM PMSF to inhibit protease activity. The crude lysate was dissolved in a buffer containing 20 mM Tris pH 8.0, 20 mM imidazole, and 500 mM NaCl, followed by centrifugation at 15,000 rpm for 60 min. The supernatant was subjected to nickel affinity chromatography, with a gradient elution using 20–300 mM imidazole. Following elution, the samples underwent dialysis with TEV protease cleavage for 72 h at 4 °C in a buffer containing 20 mM Tris pH 7.5, 50 mM NaCl, and 3 mM *β*-mercaptoethanol. The TEV protease was subsequently removed using nickel affinity chromatography, and further purification was carried out using angel filtration chromatography system with a buffer containing 20 mM Tris pH 7.5, 150 mM NaCl, and 3 mM *β*-mercaptoethanol. Homogeneous fractions were collected, concentrated to 20 mg/mL, flash-frozen in liquid nitrogen, and stored at −80 °C.

### 4.2. Crystallization of Cyt c6

Initial crystallization screening was conducted using commercially available kits containing (NH_4_)_2_SO_4_ (QIAGEN and Hampton), employing the sitting-drop diffusion for both Cyt c6 variants. The oxidized form of Cyt c6 was dissolved in buffer containing a 40-fold molar excess of potassium ferricyanide and crystals were grown in this oxidizing buffer. The reduced forms of Cyt c6 were prepared in a solution containing 3 mM *β*-mercaptoethanol. Detailed crystallization conditions and methods are detailed in Table S1.

### 4.3. Data Collection, Processing, and Refinement

Diffraction data were collected at the Shanghai Synchrotron Radiation Facility (SSRF) using beamlines SSRF-BL19U1 (red-Cyt c6-7942), SSRF-BL18U1 (red-Cyt c6-6803) and SSRF-BL02U1 (oxi-Cyt c6-7942). Data indexing, integration and scaling were performed using XDS [37]. Molecular replacement was carried out using Phaser ver. 2.8 from the CCP4 suite ver. 7.0 [38], with the crystal structure of the Cyt c6 Q57V mutant from *Synechococcus* sp. PCC 7002 (PDB code: 4EID) as the initial model. Structural refinement was performed using Phenix.refine ver. 1.19 [39] and Refmac ver. 5.8 [40], and model building was carried out with Coot ver. 0.8.9 [41]. The quality of the final structures was assessed using MolProbity ver. 4.5 [42]. Statistics for data collection and refinement are provided in Table S2. Figures were generated using PyMOL ver. 3.0 (Schrödinger, New York, NY, USA). The structural superposition and the RMSD calculation was performed using PyMOL ver. 3.0 and the LSQAB program within the CCP4 suite ver. 7.0.

### 4.4. MALDI-TOF MS Analysis

For mass spectrometry, both Cyt c6 variants were prepared in a buffer containing 20 mM Tris pH 7.5, 150 mM NaCl, and 3 mM *β*-mercaptoethanol, with a protein concentration adjusted to approximately 1 mg/mL. The samples were desalted and analyzed using CHCA as the matrix. Mass spectra were acquired using an UltrafleXtreme mass spectrometer (Bruker Daltonics, Bremen, Germany), controlled by FlexControl 3.4 software. The instrument was externally calibrated using the Bruker protein calibration kit, and spectra were recorded in the *m*/*z* range of 700 to 15,000 in linear positive ion mode (laser intensity 95%, ion source 1 = 20.00 kV, ion source 2 = 18.85 kV, lens = 5.62 kV, detector voltage = 2924 V, pulsed ion extraction = 270 ns). Each spectrum represented the accumulation of 2000–5000 laser shots across the sample spot. The spectra were processed using FlexAnalysis v.3.4 software (Bruker Daltonics) with default parameters.

### 4.5. UV–Visible Absorption Spectroscopy

The concentrations of both Cyt c6 variants were adjusted to 1 mg/mL in a buffer containing 20 mM Tris pH 7.5 and 150 mM NaCl. Oxidation of Cyt c6 was achieved by dissolving the protein in an additional buffer containing a 40-fold molar excess of potassium ferricyanide. Following oxidation, the protein was buffer-exchanged into a solution of 20 mM Tris pH 7.5 and 150 mM NaCl. UV-visible absorption spectra were recorded using a Shimadzu UV2600 spectrophotometer (Shimadzu Corporation, Kyoto, Japan). Baseline corrections were performed using the buffer as a reference, and spectra were scanned from 250 to 650 nm. Data were normalized and plotted using Origin 2021 software.

### 4.6. AlphaFold3 Prediction

AlphaFold3 (https://alphafoldserver.com/, accessed on 12 November 2024) was used to predict the PSI-Cyt c6 complex structure from *S. elongatus* PCC 7942 [28]. The Cyt c6 amino acid sequence was retrieved from UniProt website (https://www.uniprot.org/ accessed on 11 November 2024) (UniProt ID: P25935), while the PSI sequences were obtained from the PDB (PDB code: 6KIF) [29], excluding the antennae sequences and retaining only the core PSI subunits, including PsaA, PsaB, PsaC, PsaD, PsaE, PsaF, PsaI, PsaJ, PsaK, PsaL, and PsaM. A heme molecule was also incorporated as a ligand during the prediction. PyMOL ver. 3.0 (Schrödinger, New York, NY, USA) was used for visualization and analysis, revealing interaction details and potential electron transfer pathways between PSI and Cyt c6.

## 5. Conclusions

In this study, we determined the high-resolution crystal structures of Cyt c6 from *S. elongatus* PCC 7942 and *S*. PCC 6803, revealing conserved dimerization patterns around the heme crevice that are stabilized by weak hydrophobic interactions. Our findings indicate that Cyt c6 dimerization enhances protein stability, optimizes electron transfer kinetics, and provides resilience against oxidative stress, contributing to cyanobacterial adaptation to extreme environments. Using AlphaFold3, we predicted the structure of the PSI-Cyt c6 complex, shedding light on the molecular interactions involved in electron transfer. These insights underscore the versatility, flexibility, and rapid efficiency of Cyt c6 as an electron carrier, which is crucial for maintaining photosynthetic efficiency. Future research should focus on elucidating the functional implications of Cyt c6 dimerization and its interaction with PSI through mutagenesis, cross-linking experiments, Co-IP and in vivo studies, ultimately enhancing our understanding of photosynthetic mechanisms and advancing biohybrid energy system development.

## Data Availability

The atomic coordinates and structure factors have been deposited in the Protein Data Bank under accession codes 9KRD (red-Cyt c6-7942), 9KRR (red-Cyt c6-6803) and 9KRC (oxi-Cyt c6-7942).

## Supplementary Materials

The following supporting information can be downloaded at: https://www.mdpi.com/article/10.3390/ijms26020824/s1.
