# Supplementary material for: A High-Resolution Crystallographic Study of Cytochrome c6: Structural Basis for Electron Transfer in Cyanobacterial Photosynthesis
Source: Int J Mol Sci. 2025 Jan 19;26(2):824. doi: 10.3390/ijms26020824 (PMC11765882; doi:10.3390/ijms26020824)
Supplement: Supplementary file 1 [file ijms-26-00824-s001.zip › ijms-3373209-supplementary.pdf]

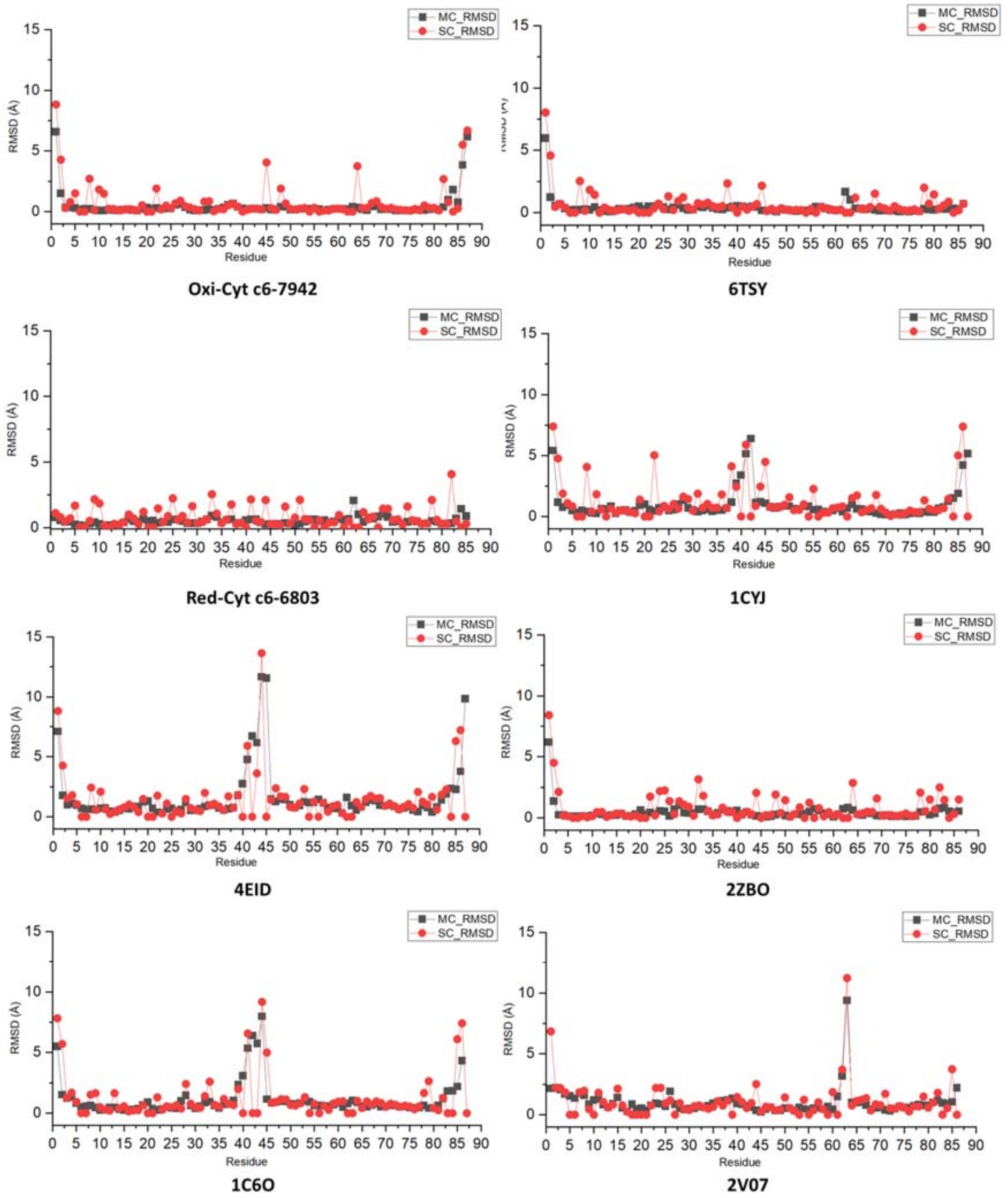

Figure S1. RMSD per residue diagrams for different species. They were generated by comparing their structures to the red-Cyt c6-7942 structure, using the LSQAB program within the CCP4 suite. MC represents the main chain, while SC represents the side chain.

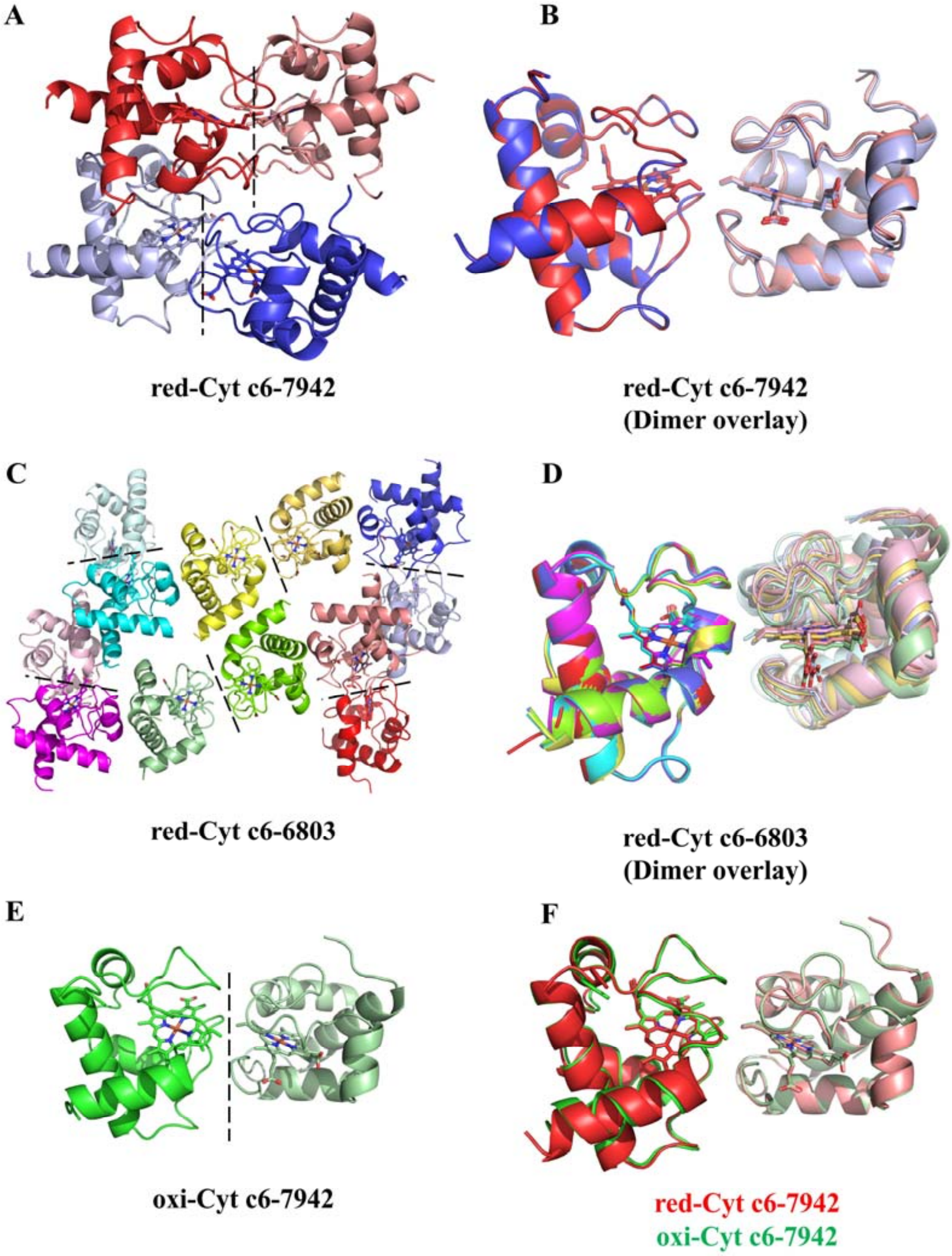

Figure S2. Comparison of dimer structures in crystals. (A) In the crystal structure of red-Cyt c6-7942, four protein molecules are present in the asymmetric unit, forming two dimers. Dashed lines indicate the interfaces in dimers. (B) The dimers extracted from the asymmetric unit of red-Cyt c6-7942 are superimposed. (C) In the crystal structure of red-Cyt c6-6803, twelve protein molecules are present in the asymmetric unit, forming six dimers. (D) The dimers extracted from the asymmetric unit of red-Cyt c6-6803 are superimposed. (E) In the crystal structure of oxi-Cyt c6-7942, two protein molecules are present in the asymmetric unit, forming one dimer. (F) Structural alignment of the dimers from red-Cyt c6-7942 and oxi-Cyt c6-7942.

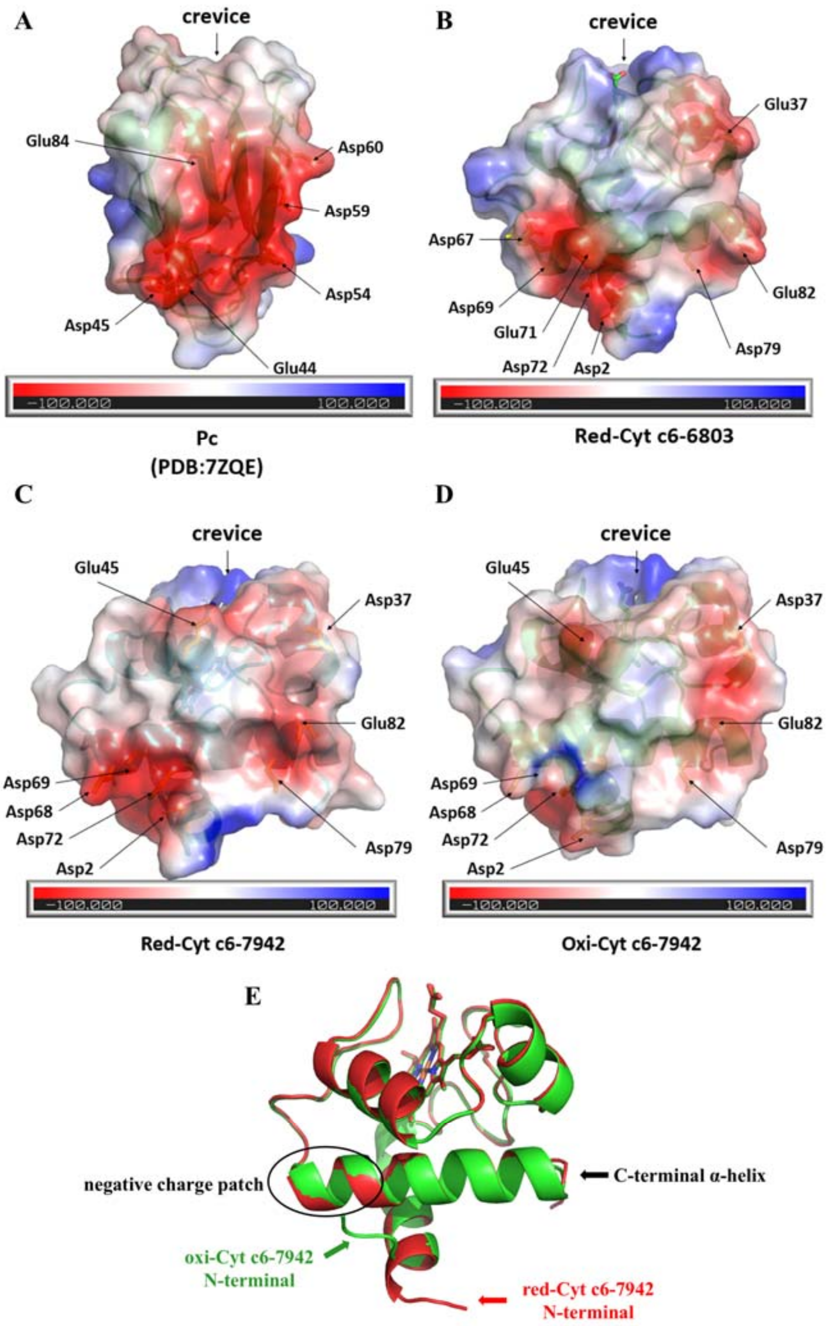

Figure S3. The surface charge distributions of Pc from *Chlamydomonas reinhardtii* (PDB: 7ZQE) (A), red-Cyt c6-6803 (B), red-Cyt c6-7942 (C), and oxi-Cyt c6-7942 (D). (A-D) The surfaces are annotated with negatively charged amino acid residues. Arrows indicate the primary distribution of negatively charged amino acids on the surfaces. Potentials are colored red and blue for negative and positive charges. (E) Structural alignment of red-Cyt c6-7942 (red color) and oxi-Cyt c6-7942 (green color) , with arrows indicating the structural differences at the N-terminus. The structural positioning in panel E is the same as in panels A-D.

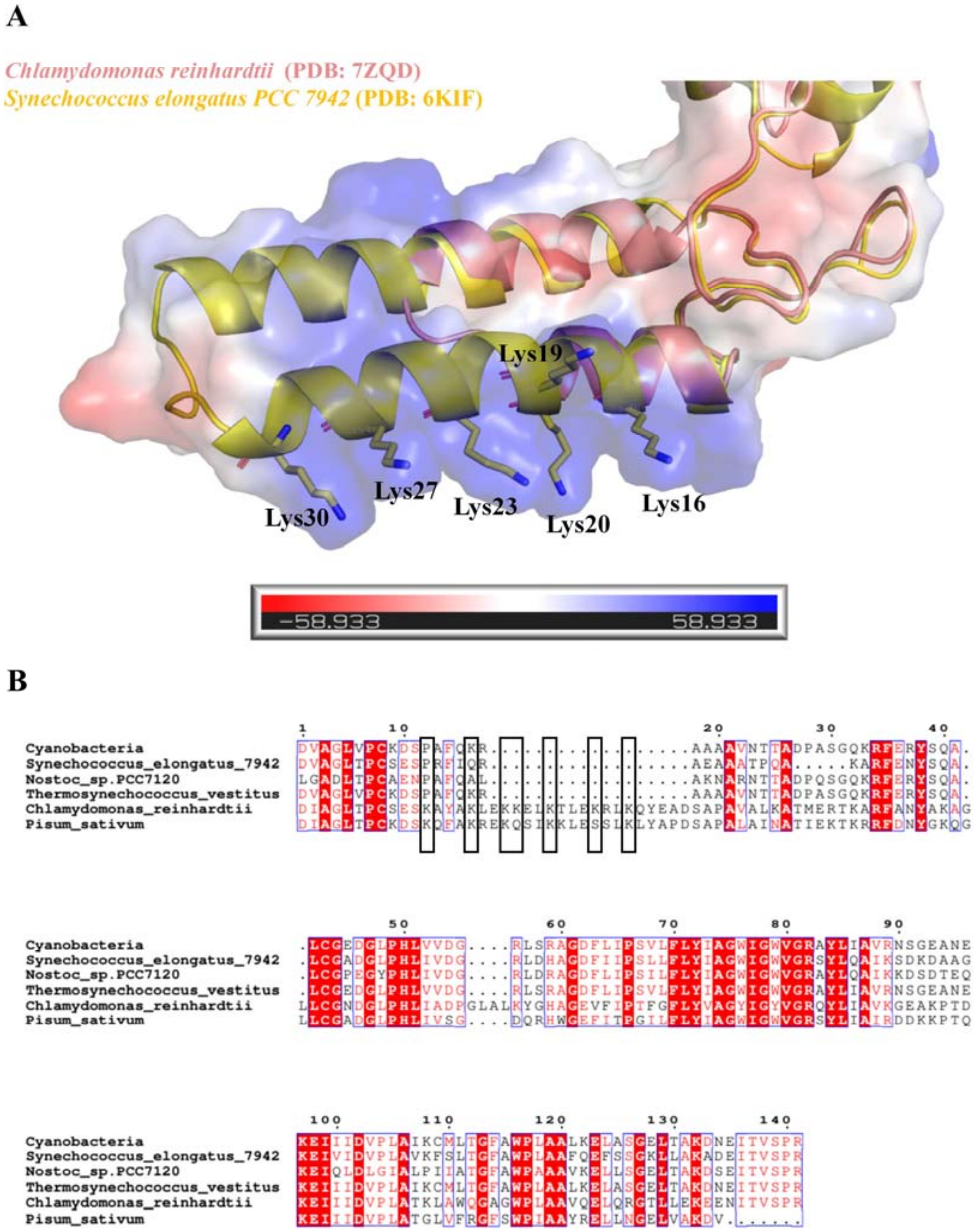

Figure S4. Comparative analysis of PsaF subunits. (A) Structural comparison of the PsaF subunits from PSI of *Chlamydomonas reinhardtii* (PDB code: 7ZQD) and *S. elongatus* PCC 7942 (PDB: 6KIF) is shown. The electrostatic surface potentials of the structure (PDB code: 7ZQD) are shown, with negative and positive charges colored red and blue, respectively. The key lysine residues involved in PSI's recognition of electron donors are depicted as sticks. (B) Sequence alignment. Lysine residues on PsaF that interact with Pc are enclosed in black boxes.

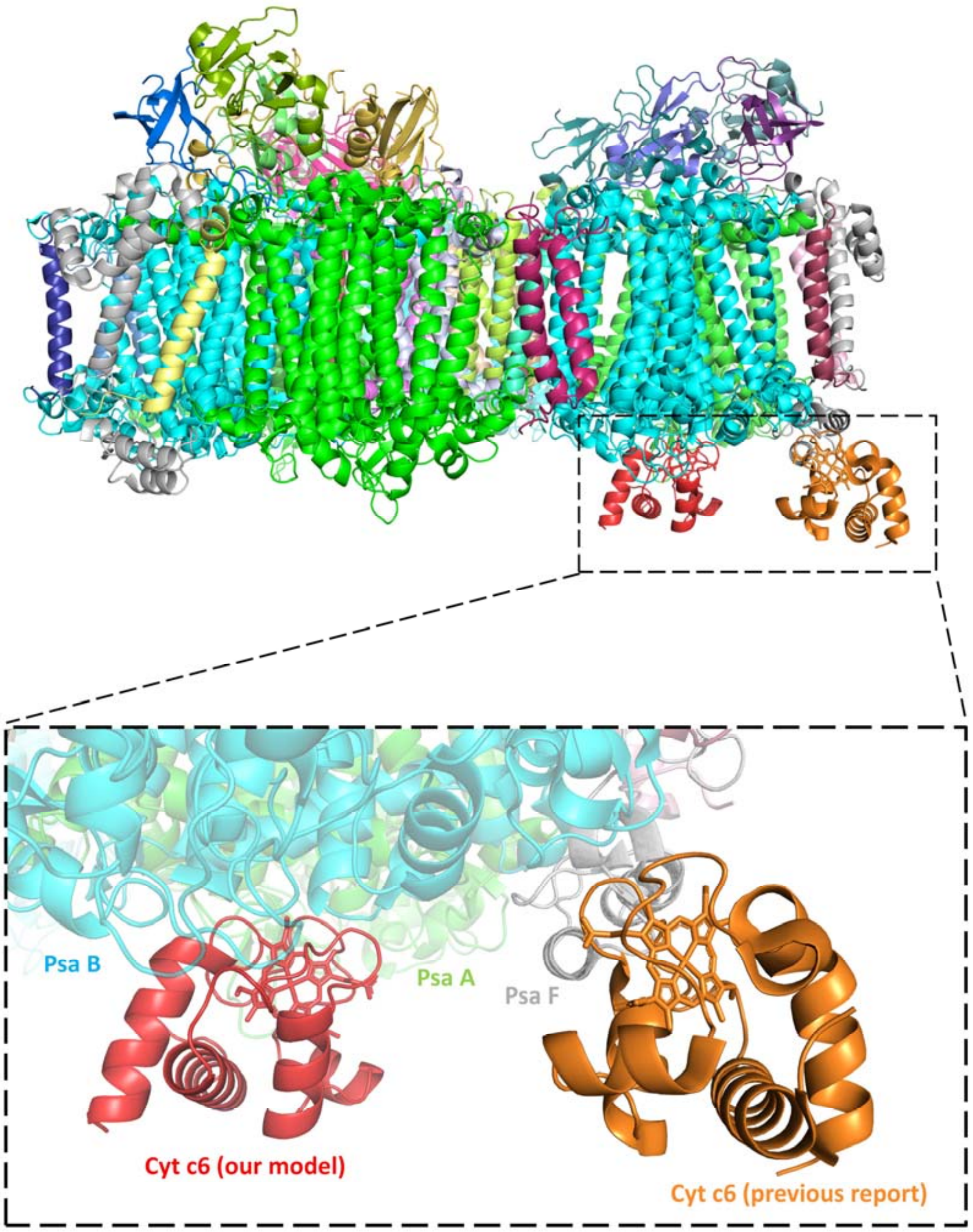

Figure S5. Comparison of our predicted PSI-Cyt c6 model with previously reported potential Cyt c6 binding sites in electron microscopy (EM) studies. Using the EM density map from earlier research (EMD code: 31605), we fitted one Cyt c6 structure into the density at a relatively low threshold to visualize possible binding locations. The fitted Cyt c6 structure is shown in orange, and our predicted binding site is shown in red. The PSI subunits are colored as same as Fig. 5.

Table S1：Crystallization conditions.

	**red-Cyt c6-7942**	**red-Cyt c6-6803**	**oxi-Cyt c6-7942**
Method	Vapour diffusion, sitting drop	Vapour diffusion, sitting drop	Vapour diffusion, sitting drop
Plate type	48-well plate	48-well plate	48-well plate
Temperature (K)	294	294	294
Protein concentration (mg ml^{-1})	20	15	10
Buffer composition of protein solution	20 mM Tris pH 7.5, 150 mM Nacl, 3 mM β-mercaptoethanol	20 mM Tris pH 7.5, 150 mM Nacl, 3 mM β-mercaptoethanol	20 mM Tris pH 7.5, 150 mM Nacl, 2 mM potassium ferrocyanide
Composition of reservoir solution	0.1 M Citric acid pH 4.0, 1.6 M Ammonium sulfate	0.2 M Sodium iodide, 2.2 M Ammonium sulfate	0.2 M di-Ammonium tartrate, 2.2 M Ammonium sulfate
Drop volume and mixing ratio	0.7 µl protein solution, 0.7 µl reservoir solution	0.7 µl protein solution, 0.7 µl reservoir solution	1 µl protein solution, 1 µl reservoir solution
Volume of reservoir (µl)	100	100	100

Table S2： Statistics for data collection and refinement.

	**red-Cyt c6-7942**	**red-Cyt c6-6803**	**oxi-Cyt c6-7942**
**Data collection**			
Diffraction source	SSRF-BL19U1	SSRF-BL18U1	SSRF-BL02U1
Detector	Pilatus3 S 6M	Pilatus3 S 6M	DECTRIS EIGER2 S 9M
Wavelength (Å)	0.98	0.98	0.98
Space group	$P2_12_12_1$	$P2_1$	$P2_12_12_1$
**Cell dimensions**			
a, b, c (Å)	54.5, 73.9, 98.8	74.9, 74.4, 133.2	50.4, 53.5, 65.9
α, β, γ (°)	90, 90, 90	90, 106.1, 90	90, 90, 90
Resolution (Å)	50-1.7 (1.76-1.70)[a]	50-1.9 (2.04-1.93)[a]	50-1.4 (1.45-1.40)[a]
R_{merge}	0.049 (0.237)	0.072 (0.494)	0.028 (0.147)
R_{pim}	0.016 (0.085)	0.036 (0.262)	0.020 (0.104)
$I/\sigma I$	29.2 (7.2)	12.6 (3.0)	21.8 (5.6)
Molecules per ASU	4	12	2
Completeness (%)	96.9 (83.2)	99.8 (98.9)	99.2 (92.8)
Redundancy	10.0 (8.4)	5.6 (5.0)	2.0 (2.0)
B factor from Wilson plot (Å^2)	16.9	19.3	11.6
**Refinement**			
Resolution (Å)	20-1.7	50-1.9	42-1.4
R_{work}/R_{free}	0.167/0.187	0.168/0.210	0.144/0.170
No. reflections	43,069	102,048	35,713
**No. atoms**			
Protein	2,543	7,376	1,287
Ligand/ion	187	652	101
Water	488	1,283	313
B-factor	23.7	24.0	16.8
**R.m.s deviations**			
Bond lengths (Å)	0.008	0.007	0.008
Bond angles (°)	0.894	0.888	0.957
**Poor rotamers (%)**	0	0.1	0
**Ramachandran plot**			
Favored (%)	96.5	96.1	94.8
Allowed (%)	3.5	3.9	4.6
Disallowed (%)	0	0	0.6
**PDB code**	9KRD	9KRR	9KRC

[a] The values in parenthesis mean those of the highest resolution shell.

Table S3：RMSD analysis of Cyt c6 monomers compared to red-Cyt c6-7942.

PDB code	Organism	Global RMSD
oxi-Cyt c6-7942	*Synechococcus elongatus* sp. PCC 7942	0.210
red-Cyt c6-6803	*Synechocystis* sp. PCC 6803	0.491
4EID	*Picosynechococcus* sp. PCC 7002	0.508
1C6O	*Tetradesmus obliquus*	0.589
6TSY	*Thermosynechococcus vestitus* BP-1	0.307
1CYJ	*Chlamydomonas reinhardtii*	0.549
2ZBO	*Sargassum fusiforme*	0.336
2V07	*Arabidopsis thaliana*	0.648

Table S4：RMSD analysis of Cyt c6 dimers compared to red-Cyt c6-7942.

PDB code	Organism	Global RMSD
oxi-Cyt c6-7942	*Synechococcus elongatus* sp. PCC 7942	0.446
red-Cyt c6-6803	*Synechocystis* sp. PCC 6803	2.415
1C6O	*Tetradesmus obliquus*	2.899
6TSY	*Thermosynechococcus vestitus* BP-1	0.752
2V07	*Arabidopsis thaliana*	12.643
